# Combining Bioinformatics and Experiments to Identify *CREB1* as a Key Regulator in Senescent Granulosa Cells

**DOI:** 10.3390/diagnostics10050295

**Published:** 2020-05-11

**Authors:** Pei-Hsuan Lin, Li-Te Lin, Chia-Jung Li, Pei-Gang Kao, Hsiao-Wen Tsai, San-Nung Chen, Zhi-Hong Wen, Peng-Hui Wang, Kuan-Hao Tsui

**Affiliations:** 1 Department of Obstetrics and Gynecology, Kaohsiung Veterans General Hospital, Kaohsiung 813, Taiwan; peihsuan0308@gmail.com (P.-H.L.); litelin1982@gmail.com (L.-T.L.); nigel6761@gmail.com (C.-J.L.); peipei5123@gmail.com (P.-G.K.); drtsai0627@gmail.com (H.-W.T.); snchen@vghks.gov.tw (S.-N.C.); 2 Daan Maternal and Children Hospital, Tainan 700, Taiwan; 3 Institute of BioPharmaceutical Sciences, National Sun Yat-sen University, Kaohsiung 804, Taiwan; 4 Department of Obstetrics and Gynecology, School of Medicine, National Yang-Ming University, Taipei 112, Taiwan; phwang@vghtpe.gov.tw; 5 Department of Marine Biotechnology and Resources, National Sun Yat-sen University, Kaohsiung 804, Taiwan; wzh@mail.nsysu.edu.tw; 6 Department of Obstetrics and Gynecology, Taipei Veterans General Hospital, Taipei 112, Taiwan; 7 Department of Medical Research, China Medical University Hospital, Taichung 404, Taiwan; 8 Female Cancer Foundation, Taipei 104, Taiwan; 9 Department of Pharmacy and Master Program, College of Pharmacy and Health Care, Tajen University, Pingtung County 907, Taiwan

**Keywords:** aging, bioinformation, biogenesis, mitochondria, oxidative stress

## Abstract

Aging of functional ovaries occurs many years before aging of other organs in the female body. In recent years, a greater number of women continue to postpone their pregnancies to later stages in their lives, raising concerns of the effect of ovarian aging. Mitochondria play an important role in the connection between the aging granulosa cells and oocytes. However, the underlying mechanisms of mitochondrial dysfunction in these cells remain poorly understood. Therefore, we evaluated the molecular mechanism of the aging granulosa cells, including aspects such as accumulation of mitochondrial reactive oxygen species, reduction of mtDNA, imbalance of mitochondrial dynamics, and diminished cell proliferation. Here, we applied bioinformatics approaches, and integrated publicly available resources, to investigate the role of *CREB1* gene expression in reproduction. Senescence hallmark enrichment and pathway analysis suggested that the downregulation of bioenergetic-related genes in *CREB1*. Gene expression analyses showed alterations in genes related to energy metabolism and ROS production in ovary tissue. We also demonstrate that the biogenesis of aging granulosa cells is subject to *CREB1* binding to the *PRKAA1* and *PRKAA2* upstream promoters. In addition, cofactors that regulate biogenesis significantly increase the levels of *SIRT1* and *PPARGC1A* mRNA in the aging granulosa cells. These findings demonstrate that *CREB1* elevates an oxidative stress-induced senescence in granulosa cells by reducing the mitochondrial function.

## 1. Introduction

Ovarian aging is one of the earliest signs of aging in the female body, and has become a cause for concern as more women postpone their childbearing age in modern society. The main cause for the decline in female fertility is the decrease in the number of oocytes, the decline in oocyte quality, and the reduction in hormone levels during ovarian aging, which in turn leads to diseases of the female reproductive system [1,2].

Within the ovarian follicle, the fitness of the oocyte is maintained by a bidirectional signaling between the oocyte and the surrounding granulosa cells [3]. In particular, the cumulus-oocyte complexes coordinate energy metabolism to provide oocytes with sufficient energy to undergo meiosis and support an embryonic development [1,4,5]. Granulosa cells metabolize glucose in the bloodstream to pyruvate and then supply it to the oocytes to enable oxidative phosphorylation to produce ATP [6,7]. The granulosa cells and the oocyte mitochondria are the major mediators of these metabolic pathways and are directly involved in the establishment of oocyte quality during oogenesis [8,9].

During an aerobic metabolism, the process of generation of adenosine triphosphate (ATP) and oxidative phosphorylation inevitably produces reactive oxygen species (ROS) [10]. These include superoxide anion radicals, hydroxyl radicals, and hydrogen peroxide, which can destroy biological molecules and endanger self-regulation mechanisms [11]. Exposure to ROS leads to the deterioration of oocyte quality, rapid consumption of the follicles, and a decrease in the mitochondrial electron transport chain activity in the oocytes [12]. As the mitochondrial respiratory chain complexes age, the complex activity reduces, resulting in a blocked electron transfer, an ineffective oxygen use, and the generation of a large amount of oxygen free radicals [13].

Organisms produce energy through mitochondria to provide homeostasis for cells. At the molecular level, many transcription factors and cofactors are involved in the activation and regulation of mitochondrial biogenesis. The CREB expression level is directly related to the mitochondrial biogenic activity. The activation of the transcription factor *CREB1* mediates the activities of PGC1A, AMPK and ATF2, or regulates the phosphorylation of CREB by PKA [14,15,16]. Therefore, CREB plays the core role of biogenesis and activates the regulatory component of mitochondrial biogenesis, which has become a promising research field for enhancing geriatrics.

In the later stages of female reproductive life, the remaining oocytes in the ovaries are maintained in a quiescent state before ovulation [17]. The increase in female age and the extended dormancy of the ovaries also results in an ROS accumulation [18]. The accumulation of oxidized products causes deletion mutations in the mitochondrial DNA (mtDNA) to continuously accumulate [19]. This seriously affects the mitochondrial functions as the respiratory chain complex activity and ATP synthesis are further reduced and ROS continues to increase [12]. At the same time, the antioxidant enzyme activity in the mitochondria also decreases with age [20].

The granulosa cell microenvironment is similar to that of oocytes, and the function and activity of granulosa cells are good indicators of oocyte quality and can be used to evaluate the effects of aging on the oocytes [21,22]. Therefore, we investigated age-related changes in the mitochondria in different aspects of the granulosa cells. Based on our results, the decline in the oxidative phosphorylation function of granulosa cells due to aging is more significant than the mitochondrial copy number and genetic integrity, indicating ovarian aging and reveals strategies to improve the assisted reproductive technology (ART) outcomes in older women. A comprehensive understanding of the underlying mechanisms of infertility related to ovarian aging will help in the better management of the disease in the future.

## 2. Results

### 2.1. Predicted Function and Pathway Enrichment Analysis 

**Figure 1:**
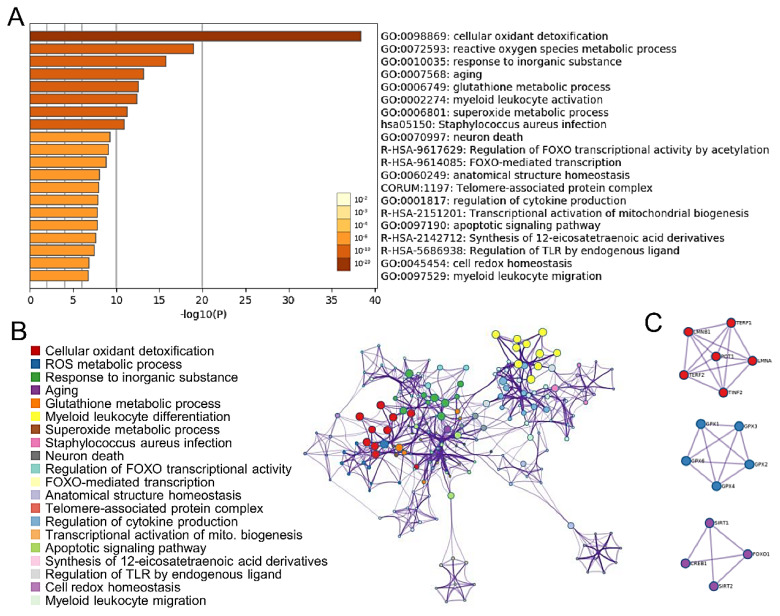
Functional enrichment and pathway analysis. (**A**) The Metascape analysis shows the top 20 clusters of enriched sets. Left panel, heatmap of the 20 enriched terms. (**B**) Representative Molecular Complex Detection (MCODE) network node showing DEG regulated by *CREB1*. (**C**) Representative Molecular Complex Detection (MCODE) network nodes, showing the *CREB1*-regulated DEGs densely connected.

To investigate the biological significance of these overlapping genes, we uploaded the list of 96 upregulated genes into Metascape software for functional enrichment analysis. The Metascape analysis shows the top 20 clusters of enriched sets (Figure 1A). These genes were enriched in the molecular function categories telomere-associated protein complex, aging, and mitochondrion organization. To further capture the associations that exist between terms, a subset of rich terms was selected and presented as a network graph. Subsequently, we chose the term with the best p-value. Metascape was used to construct the protein-protein interaction (PPI) network of the 96 upregulated genes (Figure 1B). The network was visualized using Cytoscape, where each node represents a rich term and is first colored according to its cluster ID (Figure 1C). 

### 2.2. Establishment and Characteristics of the Aged Granulosa Cells

**Figure 2:**
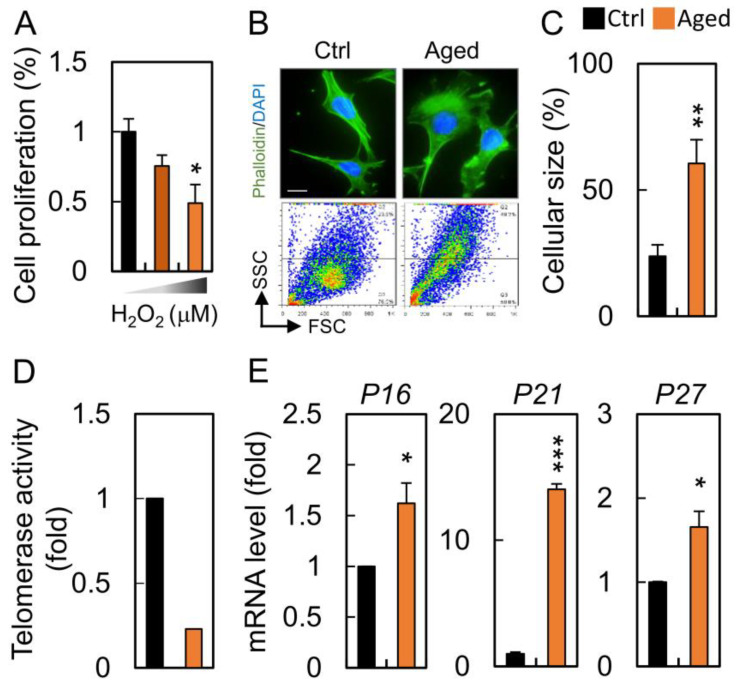
Granulosa cells undergo senescence after hydrogen peroxide treatment. (**A**) HGL5 cells were treated with different concentrations of H_2_O_2_ for 24 h, and the cell proliferation was assessed by CCK8. (**B**) The cells were stained for cytoskeleton and analyzed for cellular granularity during senescence. (**C**) Quantification of cellular size was determined between control and aged groups. (**D**) Determination of telomerase activity by qPCR. (**E**) The expression of senescence markers in control and senescent cells. Scare bar = 20 µm, * *p* < 0.05, ** *p* < 0.01, and *** *p* < 0.001.

To ascertain the effect of hydrogen peroxide on cellular senescence, HGL5 cells were incubated with hydrogen peroxide. HGL5 cells were sensitive to increasing concentrations of H_2_O_2_ with an IC_50_ value of 100 μM at 24 h (Figure 2A). Senescent granulosa cells exhibited typical morphological changes characteristic of aged cells as they became flattened and enlarged when compared to those in the control groups (Figure 2B). We also observed that the senescent phenotype gradually developed with H_2_O_2_ treatment, which was confirmed by an increase in the cellular granularity, cell size and telomerase activity (Figure 2C,D). To further validate our model of cellular aging, we determined that mRNA expression for the senescence biomarkers, *P16*, *P21* and *P27*, was significantly increased during cell senescence (Figure 2E).

### 2.3. Mitochondrial Dysfunction in Granulosa Cells Undergoing Senescence

**Figure 3:**
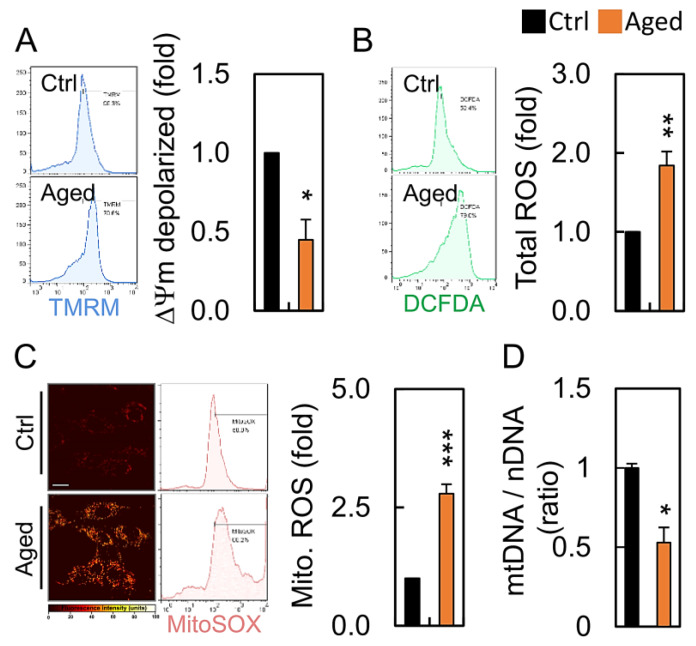
Aging leads to mitochondrial dysfunction: (**A**) mitochondrial membrane potential, (**B**) cellular ROS levels, (**C**) mitochondrial ROS, and (**D**) mitochondrial DNA content in in HGL5 cells treated with or without hydrogen peroxide. * *p* < 0.05, ** *p* < 0.01, and *** *p* < 0.001.

To further examine whether mitochondrial dysfunction was present in the granulosa cells undergoing senescence, the overall mitochondrial function of the aged granulosa cells was measured at 24 h. The mitochondrial membrane potential (MMP) analysis revealed that H_2_O_2_ resulted in a dramatic reduction of the membrane potential depolarization, indicating a loss of MMP and the damage of mitochondria in the aged granulosa cells (Figure 3A). The total cellular and mitochondrial ROS of the H_2_O_2_-treated HGL5 was higher than that of the nontreated control group (Figure 3B,C). A lack of mitochondrial sufficiency in the senescent cells also led to a marked reduction in the mtDNA copy number. (Figure 3D).

### 2.4. Abnormal Mitochondrial Dynamics in Aged Granulosa Cells

**Figure 4:**
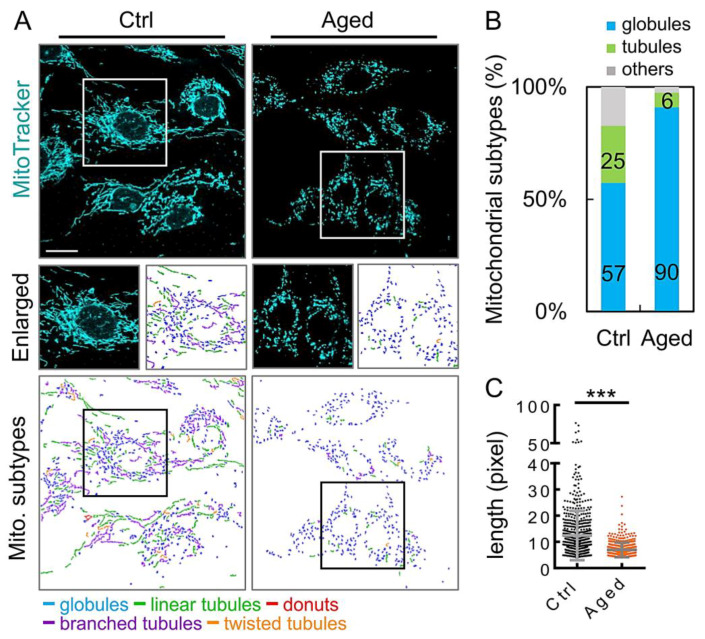
Aging granulosa cells exhibit increased fragmentation. (**A**) Mitochondria in granulosa cells, with or without H_2_O_2_ treatment, were labeled with MitoTracker. (**B**) Three major types of mitochondria were quantified: globules, tubules, and others. (**C**) The total length of each mitochondrion was determined. Scare bar = 20 µm, *** *p* < 0.001.

To assess whether the mitochondrial network of the aging granulosa cells was imbalanced, we analyzed changes in the mitochondrial morphology. The mitochondria were classified into three types according to their morphological characteristics (globules, tubules, and others), using the MicroP software [23]. After tracking mitochondria with fluorescent dyes, we found that mitochondrial elongation in the aging group was significantly reduced (Figure 4A). Moreover, the percentage of fragmented mitochondria shown by the aging group was significantly higher than that of the control group (Figure 4B). In addition, the average mitochondrial length was significantly reduced in the aged group compared to that in the control group (Figure 4C).

**Figure 5:**
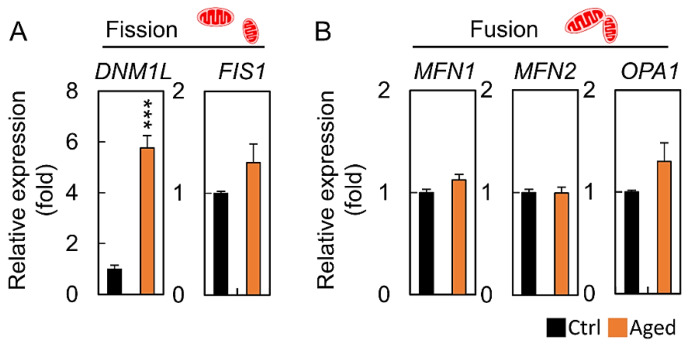
Aging leads to mitochondrial dynamics imbalance and increased *DNM1L* expression. (**A**,**B**) Quantitative real-time polymerase chain reaction analysis for mRNA expression of mitochondrial dynamics-related genes of granulosa cells among the control and aged groups. All values are normalized to *GAPDH* and expressed as a fold of control. *** *p* < 0.001.

To explore the mechanisms underlying the observed reduction in the mitochondrial morphological change, the relative expression of the mitochondrial dynamic genes was analyzed using qRT-PCR. The mRNA levels of *DNM1L* was significantly higher in the aged group than that in the control group (Figure 5A). However, there were no significant differences in the *MFN1, MFN2, OPA1, and FIS1* expression levels between the control and the aged groups (Figure 5B).

### 2.5. Bioinformatic Analysis of CREB1 Function in Ovary

**Figure 6:**
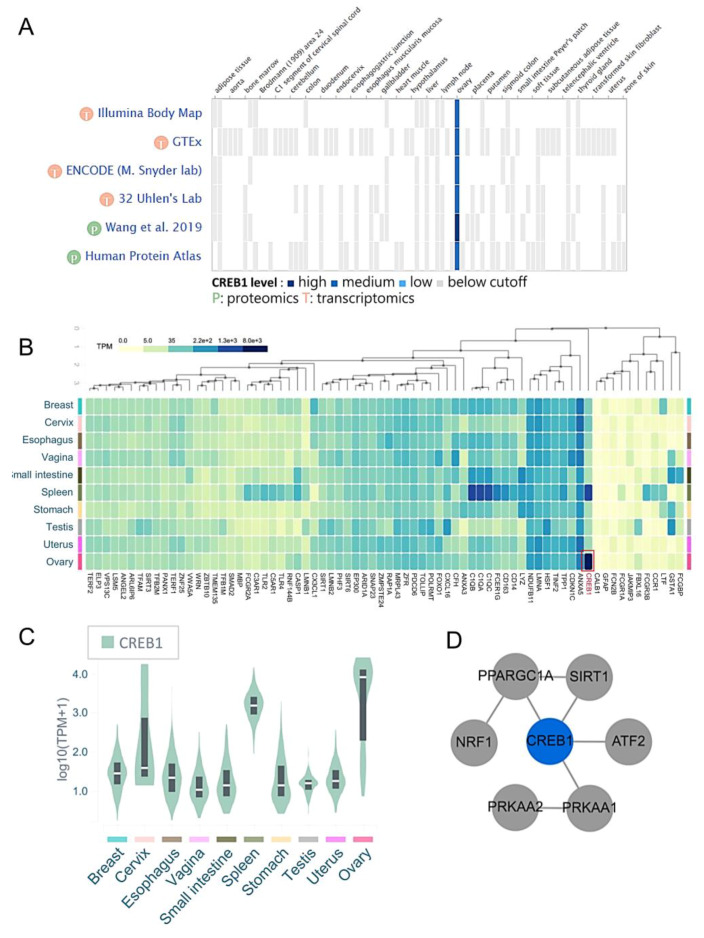
The level of *CREB1* expression in different tissues. (**A**) *CREB1* gene was analyzed for gene distribution and expression in different tissues using GTExPortal website. (**B**) Heatmap representing the *CREB1* indexes of all the 72 genes across all the tissues from Web-based Gene Set analysis Toolkit. (**C**) The *CREB1* mRNA levels in 10 different types of tissues. (**D**) The protein–protein interaction networks of genes associated with *CREB1*.

Heat maps of gene expression in the ovary from public databases show that *CREB1* mRNA expression in ovarian tissue is higher than other normal tissues (Figure 6A). In addition to ovarian tissue, the expression of *CREB1* mRNA in different types of tissues was further tested, and it was found that the expression of *CREB1* mRNA in spleen tissues is usually higher (Figure 6B). In addition, compared with other genes, it was further confirmed that the expression of *CREB1* was significantly up-regulated in ovarian tissue (Figure 6C). The enriched terms included the glutathione metabolism, ferroptosis, *CREB1* regulates biogenesis genes, and positive regulation of cellular senescence. Meanwhile, the network of core modules of genes (*PPARGC1A, SIRT1, ATF2, NRF1, PRKAA1, PRKAA2*), as well as core enriched term linked to *CREB1*, were also constructed by String, which indicating important and potential biomarkers that contributed to the development and progression of senescence with *CREB1* (Figure 6D).

### 2.6. Regulation of the CREB/PRKAA Transcription Factor Axis in Aged Granulosa Cells

**Figure 7:**
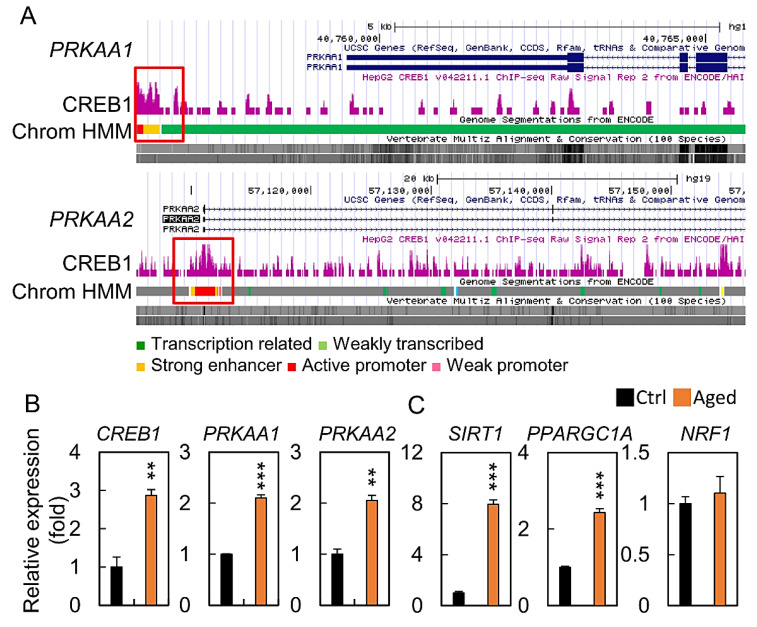
Age-related changes in key metabolic and transcriptional regulators. (**A**) Scheme of the human genomic region encompassing the *CREB1* promoter. The purple peaks represent the promoter binding regions, according to ENCODE. The red square shows the amplified region in the promoter. (**B**,**C**) mRNA expression level of *CREB1, PRKAA1, PRKAA2, SIRT1, PPARGC1A*, and *NRF1*. All values were normalized to *GAPDH* and expressed as a fold of control. ** *p* < 0.01, and *** *p* < 0.001.

We further explored the upstream regulators that potentially mediated the decreased mitochondrial biogenesis level. Hydrogen peroxide treatment may regulate the expression of *PRKAA1* and *PRKAA2* mRNA, which are the key effectors of a mitochondrial biogenesis. Publicly available sequencing data showed that *CREB1* could bind directly to the *PRKAA1* and *PRKAA2* promoters (Figure 7A). qPCR with granulosa cells showed that endogenous *CREB1* was bound to both *PRKAA1* and *PRKAA2* during cell senescence, while treatment with H_2_O_2_ markedly elevated this (Figure 7B). We further showed that *SIRT1* and *PPARGC1A* mRNA expressions increased in the aging group compared to those in the control, while there was no significant change in the *NRF1* mRNA expression (Figure 7C).

## 3. Discussion

Mitochondria are a major factor in the oocyte quality and are vital in the supply of sufficient ATP, but these may be directly affected during ovarian aging [1,11]. Although mitochondria have been hypothesized to be involved in energy metabolism, calcium homeostasis, growth and apoptosis [12,24,25], they have also been indicated to be the main source of intracellular ROS production [26,27]. Previous studies have reported that as age increases, the oocyte mass decreases, and mitochondrial dysfunction, oocyte mtDNA mutation, and deletion levels increase [12,28]. Studies have also shown that the fertilization capacity and subsequent embryo growth potential of oocytes is directly proportional to the mtDNA content of older women, which is closely related to the ATP production in developing embryos [29]. Owing to the higher energy requirements of the developing embryos, oocyte maturation, division, preimplantation, and embryogenesis, a reduced glycolysis and preservation of the mtDNA function, prior to the oocyte blastocyst stage, provides the main source of ATP [30,31,32]. 

In the present study, we focused on the mitochondria in granulosa cells and found age-related changes in the mitochondrial morphology and functions, accompanied by a decreased MMP in the aging group. However, changes in the intracellular ROS level, the mtDNA content, and mtDNA integrity did not decline significantly with aging. Finally, a reduced mitochondrial DNA copy number and an impaired ability of mitochondrial biogenesis were observed in the study, which may be mainly responsible for the age-related dysfunction of the granulosa cells [33]. In our study, the mitochondria in the younger-age group were mostly elongated. In contrast, the mitochondria in the older-age group were fragmented and accompanied by a higher mitochondrial ROS production [10]. Intracellular ATP levels are often measured as a key indicator of the mitochondrial function; however, the view of mitochondria and reproductive aging in granulosa cells remains controversial. We hypothesized that the mtDNA content in cells would decrease with aging and ultimately regulate the homeostasis of mitochondria in the granulosa cells.

Mitochondria are highly dynamic organelles that move, fuse, and divide continuously according to changes in the cellular energy requirements. Mitochondrial dynamics are mediated by large dynamic GTPases (DRP1, FIS1, OPA1, MFN1, and MFN2) embedded in the mitochondrial membrane [34,35]. Mitochondrial division produces new organelles necessary for cell growth and cell proliferation, while encouraging the elimination of damaged mitochondria [36]. Mitochondrial fusions ensure tight complementarity between organelles to meet the energy needs at the cellular level [37]. Noteworthy, our results showed that the mitochondrial fragmentation morphology of granular cells in the aging group was as high as 90% (Figure 4B). Although the *DNM1L* mRNA level in the aging group was approximately six-fold that of the control group (Figure 5A), there were no significant differences in expression of the other genes tested. This result indicates that the DRP1 GTPase, which regulates mitochondrial fission and is encoded by the *DNM1L* gene, plays an important role in the aging granulocytes, and the mitochondrial dynamic imbalance also reflects the result of an mtDNA reduction (Figure 3D).

Many other factors play a major role in the aging process, including key genes *SIRT1, PPARGC1A, CREB1, PRKAA1*, and *PRKAA2*. CREB1 is a major regulator of biogenesis and plays a key role in ATP production, which is critical for cell survival under stress conditions [38,39,40]. In different cell types, *CREB1* activation induced by an impaired mitochondrial activity suggests that this transcription factor plays an important role in response of the adaptive cells towards a high-energy stress [41,42]. *CREB1* was shown to have regulatory binding regions for both *PRKAA1* and *PRKAA2* upstream regions that activated expression [43,44]. In addition, mRNA expression of the biogenesis cofactors *PPARGC1A* and *SIRT1* were also associated with aging. Although the molecular mechanism of *NRF1* mRNA expression is unclear, our study clearly shows that *CREB1* is involved in the regulation of the aging granulosa cells when exposed to an oxidative stress. We also observed a higher level of *CREB1* mRNA expression in the aging group. The H_2_O_2_ stimulation induced significant *PRKAA1* and *PRKAA2* mRNA expressions, oxidative stress, mitochondrial imbalance, and cell senescence in the HGL5 cells. 

## 4. Materials and Methods 

### 4.1. Cell Culture and Treatment

The human granulosa cell line HGL5 was grown in Dulbecco’s Modified Eagle Medium/Nutrient Mixture F-12 (DMEM/F12; GIBCO Invitrogen Co.) supplemented with 10% fetal bovine serum (FBS) at 37 °C in a humidified incubator containing 5% CO_2_ and 95% air. The cells cultured in a complete medium (10% FBS) were used as the control. For the senescence experiment, the cells were treated with 100 μM H_2_O_2_ for 24 h.

### 4.2. Cell Proliferation Assay

The cell viability was analyzed using the cell counting kit-8 (CCK-8), which detected the metabolic activity of the cells. At the end of the various treatments, 10 μL of the CCK-8 reagent was added to each well, and the cells were then incubated at 37 °C for 4 h. Absorbance was recorded using an ELISA microplate reader at 450 nm.

### 4.3. RT-PCR

Total RNA was extracted with REzol (Protech Technology EC, London, UK). Levels of mRNA were analyzed with SYBR green-based real-time quantitative PCR assays (Applied Biosystems, Darmstadt, Germany), with GAPDH as the reference genes in each reaction. Sequences of the primers used for qPCR assays are shown in Table S1.

### 4.4. Mitochondrial Functional Analysis (ROS and MMP)

Cells were harvested, washed, resuspended in culture medium, and stained with DCFDA (10 μM), MitoSOX (5 μM), and TMRM (500 nM) (Molecular Probes, Eugene, CA, USA) at 37 °C for 30 min. After incubation and wash, cells were analyzed by flow cytometry (FACSCalibur, BD Bioscience, San Jose, CA, USA). 

### 4.5. Fluorescent Labeling

Cells were grown on coverslips, washed with PBS, fixed with 4% paraformaldehyde for 10 min at room temperature (RT). Mitotracker and phalloidin probe were used to label mitochondria and cytoskeleton, respectively. Briefly, cells grown on coverslips were incubated with mitotracker probe (Molecular Probes Inc., Eugene, OR, USA) for 30 min at 37 °C before fixation. Mitochondrial morphology was analyzed by categorizing cells as previously described [45]. Images were acquired using EVOS M5000 imaging system (63× objective). 

### 4.6. Big Data Analytics Tools

Metascape is a free gene annotation and analysis resource that helps researcher make sense of one or multiple gene lists [46]. The Genotype-Tissue Expression (GTEx) project is an ongoing effort to build a comprehensive public resource to study tissue-specific gene expression and regulation [47]. WebGestalt integrates functional enrichment analysis and information visualization. It permits the management, information retrieval, organization, visualization and statistical analysis of large sets of genes. STRING (a search tool for searching interacting genes/proteins) is a known and predicted biological database and network resource for protein-protein interactions [48]. The STRING database contains information from many sources, including experimental data, calculation prediction methods, and public text sets. It can be accessed for free and will be updated regularly. The resource also uses many functional classification systems (such as GO, Pfam, and KEGG) to highlight the functional enrichment in the user-provided protein list [49].

### 4.7. Telomerase Activity Assay

Quantitative determination of telomerase activity was performed using Telomerase activity quantification qPCR assay kit (Sciencell research lab., Inc., Carlsbad, CA, USA) according to the manufacturer’s protocol.

### 4.8. Statistical Analysis

The data presented are the mean ± standard error of the mean (S.E.M.) from at least 3 independent experiments and were analyzed using a Student’s t-test. All calculations were performed using GraphPad Prism, 6.0. The intensity of fluorescence was quantified and analyzed using ImageJ software (NIH) and MicroP software [50]. Differences were considered significant when *p* < 0.05. 

## 5. Conclusions

**Figure 8:**
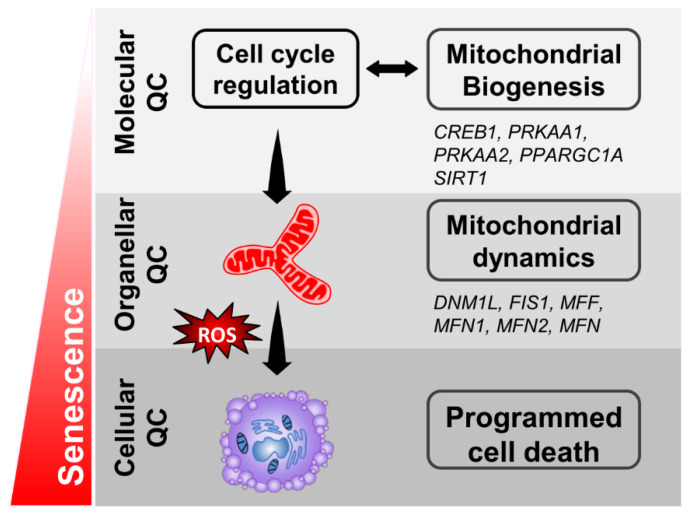
A working model of the effects in senescent granulosa cells.

In conclusion, our study demonstrates that dysfunction of the aging granulosa cells is mainly related to an impaired mitochondrial function, especially for the mitochondrial biogenesis and dynamics. Our research suggests that increasing the biogenic capacity of the granulosa cells may improve infertility in elder women receiving ART (Figure 8). In addition to the common strategies to reduce oxidative stress, preventing ovarian aging also improves the ovarian function by increasing the mitochondrial biogenesis.

## Supplementary Materials

Supplementary materials can be found at https://www.mdpi.com/2075-4418/10/5/295/s1. Table S1: Sequence of oligo-nucleotides used as RT-PCR primers.
