# Supplementary material for: Combining Bioinformatics and Experiments to Identify CREB1 as a Key Regulator in Senescent Granulosa Cells
Source: Diagnostics (Basel). 2020 May 11;10(5):295. doi: 10.3390/diagnostics10050295 (PMC7277907; doi:10.3390/diagnostics10050295)
Supplement: Supplementary file 1 [file diagnostics-10-00295-s001.pdf]

Supplementary Materials

Table S1. Sequence of oligo-nucleotides used as RT-PCR primers.

Gene	Primers
GAPDH	Forward primer: TGC ACC ACC AAC TGC TTA GC<br>Reverse primer: GGC ATG GAC TGT GGT CAT GAG
P16	Forward primer: TGTGTTGGAGTTTTCTGGAGTGA<br>Reverse primer: CAAGAAATGCCCACATGAATGT
P21	Forward primer: TGG AGA CTC TCA GGG TCG AAA A<br>Reverse primer: GCG TTT GGA GTG GTA GAA ATC TG
P27	Forward primer: AGC GCA AGT GGA ATT TCG A<br>Reverse primer: GCC ACT CGT ACT TGC CCT CTA
MFN1	Forward primer: TGT TTT GGT CGC AAA CTC TG<br>Reverse primer: TCT TTC CAT GTG CTG TCT GC
MFN2	Forward primer: ATT GCA GAG GCG GTT CGA CTC A<br>Reverse primer: TTC AGT CGG TCT TGC CGC TCT T
OPA1	Forward primer: GTG GTT GGA GAT CAG AGT GCT G<br>Reverse primer: GAG GAC CTT CAC TCA GAG TCA C
DNM1L	Forward primer: GAT GCC ATA GTT GAA GTG GTG AC<br>Reverse primer: CCA CAA GCA TCA GCA AAG TCT GG
FIS1	Forward primer: CAA GGA ACT GGA GCG GCT CAT T<br>Reverse primer: GGA CAC AGC AAG TCC GAT GAG T
mtDNA<br>mt-Co1	Forward primer: TGC TAG CCG CAG GCA TTA C<br>Reverse primer: GGG TGC CCA AAG AAT CAG AAC
nDNA<br>Ndufv1	Forward primer: CTT CCC CAC TGG CCT CAA G<br>Reverse primer: CCA AAA CCC AGT GAT CCA GC
CREB1	Forward primer: TACAAACATACCAGATTCGC<br>Reverse primer: TCCCTGTTCTTCATTAGACG
PRKAA1	Forward primer: AGGAAGAATCCTGTGACAAGCAC<br>Reverse primer: CCGATCTCTGTGGAGTAGCAGT
PRKAA2	Forward primer: CCAGAGAATGTCCTGTTGGATGC<br>Reverse primer: CTGAGATGACTTCAGGTGCTGC
SIRT1	Forward primer: TTG GCA CCG ATC CTC GAA C<br>Reverse primer: CCC AGC TCC AGT CAG AAC TAT
PPARGC1A	Forward primer: CCAAAGGATGCGCTCTCGTTCA<br>Reverse primer: CGGTGTCTGTAGTGGCTTGACT
NRF1	Forward primer: GGC AAC AGT AGC CAC ATT GGC T<br>Reverse primer: GTC GTC TGG ATG GTC ATC TCA C
